# KA-Search, a method for rapid and exhaustive sequence identity search of known antibodies

**DOI:** 10.1038/s41598-023-38108-7

**Published:** 2023-07-18

**Authors:** Tobias H. Olsen, Brennan Abanades, Iain H. Moal, Charlotte M. Deane

**Affiliations:** 1 Oxford Protein Informatics Group, Department of Statistics, University of Oxford, Oxford, OX1 3LB UK; 2 GSK Medicines Research Centre, GlaxoSmithKline plc, Stevenage, SG1 2NY UK; 3 Exscientia plc, Oxford, OX4 4GE UK

**Keywords:** Data mining, Immunology, Software

## Abstract

Antibodies with similar amino acid sequences, especially across their complementarity-determining regions, often share properties. Finding that an antibody of interest has a similar sequence to naturally expressed antibodies in healthy or diseased repertoires is a powerful approach for the prediction of antibody properties, such as immunogenicity or antigen specificity. However, as the number of available antibody sequences is now in the billions and continuing to grow, repertoire mining for similar sequences has become increasingly computationally expensive. Existing approaches are limited by either being low-throughput, non-exhaustive, not antibody specific, or only searching against entire chain sequences. Therefore, there is a need for a specialized tool, optimized for a rapid and exhaustive search of any antibody region against all known antibodies, to better utilize the full breadth of available repertoire sequences. We introduce Known Antibody Search (KA-Search), a tool that allows for the rapid search of billions of antibody variable domains by amino acid sequence identity across either the variable domain, the complementarity-determining regions, or a user defined antibody region. We show KA-Search in operation on the $\sim$2.4 billion antibody sequences available in the OAS database. KA-Search can be used to find the most similar sequences from OAS within 30 minutes and a representative subset of 10 million sequences in less than 9 seconds. We give examples of how KA-Search can be used to obtain new insights about an antibody of interest. KA-Search is freely available at https://github.com/oxpig/kasearch.

## Introduction

Antibodies have become an invaluable form of therapeutics, with an increasing number of new antibody derived therapeutics being developed and marketed each year^1^. Despite their success, the process of antibody discovery and design is still challenging^2^. Antibodies are composed of four protein chains, two identical larger chains and two identical smaller chains, called heavy and light respectively^3^. Each chain has one variable (V) domain^4^ and one or more conserved (C) domains^5^. While the C domains are encoded by a single gene, the V domain of the heavy (VH) and light (VL) chain are encoded by V and J (for light chains) and V, D and J (for heavy chains) genes, rearranged together by a process known as V(D)J recombination^6^. The V domains are then further diversified by somatic hypermutation^7,8^. The antibody binding site, i.e. the paratope, is formed by the pairing of the VH and VL, and largely consists of residues from three highly variable loops on each chain, called complementarity-determining region (CDR)1, CDR2 and CDR3^3,7,8^. Of these, the heavy chain CDR3 is the most diverse and often the main contributor to the paratope^7,8^. With the estimated number of different human antibody sequences being between $10^{16}$ and $10^{18}$, antibodies are ideal as therapeutics, as they have the potential to bind to any antigen, i.e. foreign molecules, or more specifically, any epitope, i.e. the specific region on an antigen a given paratope binds to, with a strong specificity^9^.

For the development of therapeutic antibodies, much work is done to find mutations that improve antibody binding, such as mutations in the paratope that increase the binding affinity to a desired epitope^10^. Also important are mutations that do not change binding, but instead improve other properties of potential therapeutic antibodies. This includes improving their developability profile by removing suboptimal properties, such as undesirable post-translational modification sites^11^ or potential antibody aggregation^12^. For non-human derived antibodies, issues with immunogenicity are also common, requiring mutations to make the antibody more human-like and thereby reduce the immunogenicity^13^. However, the huge space of antibody sequences and possible mutations makes it a complicated challenge to find the correct mutations to make in order to achieve the desired binding specificity and properties.

**Figure 1:**
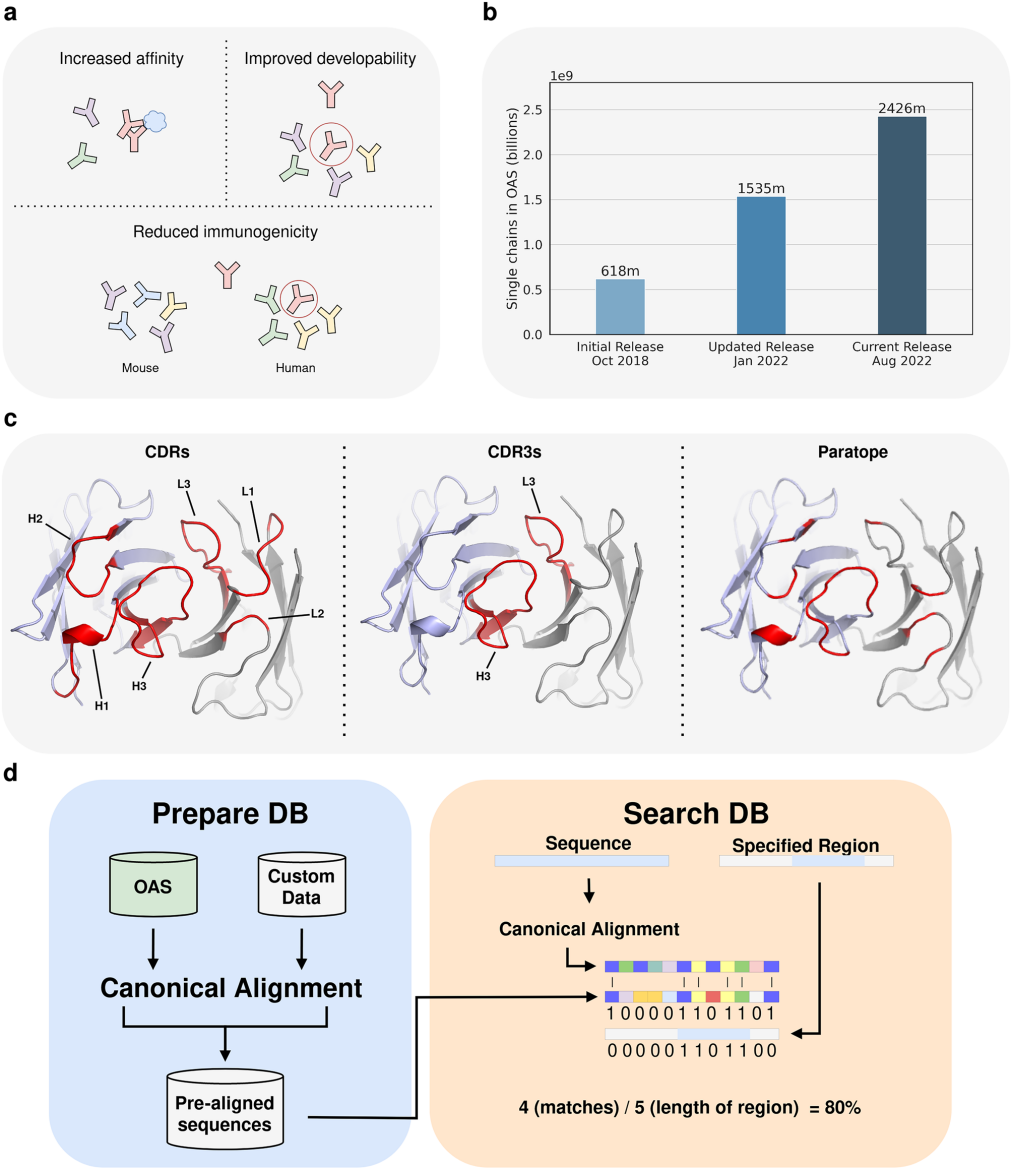
(**a**) To correctly identify relevant mutations for optimising a given antibody for increased affinity, an improved developability profile or reduced immunogenicity, a huge space of possible mutations needs to be searched. As similar antibodies often bind to the same epitope but with different strengths, immune repertoire mining can be used to find similar antibodies with potentially better binding affinity. Immune repertoire mining can also be used to find antibodies with the same binding but with mutations improving their developability profile or reducing their immunogenicity. (**b**) Overview of the available number of single chain antibody variable domain sequences in the Observed Antibody Space database over time. (**c**) Highlight (red) of different specific search regions. The antibody variable domain is derived from PDB structure 7JOO and the CDRs are annotated using IMGT numberings^37^. Heavy chain complementarity-determining region (CDR)1, 2 and 3 are denoted as H1, H2 and H3, and light chain CDRs as L1, L2 and L3. The paratope was defined as any residue within 4.5Å of the antigen (in this case an inducible T-cell costimulator) and consists of IMGT position 35, 36, 57, 58, 64, 66 and 109–113 on the heavy chain and IMGT position 37, 38, 55, 56, 66 and 114 on the light chain. **d**, Overview of KA-Search. Before search, target sequences are pre-aligned using a canonical alignment of 200 unique antibody positions. Once an antibody has been entered into a pre-aligned database this calculation does not have to be repeated even when new data is added. For search, a query and the specific region to search, for example whole variable domain or CDRs, is specified. The query is then aligned using the canonical alignment and matched with each aligned target sequence. The specific region mask is then applied before calculating the sequence identity for the region. The exact method for sequence identity calculation is either with or without length match.

A technique which has shown promise for exploring the mutational space of antibodies is immune repertoire mining^14–17^. In immune repertoire mining, an antibody of interest is compared against natural antibody repertoires to find identical or highly similar antibodies. This is useful for finding mutations which could improve binding affinity, as antibodies with few differences in the paratope, derived from patients with the disease of interest, might yield better binders. Finding antibodies in nature with an identical paratope is a powerful method for getting insight into which mutations could improve an antibody’s developability profile or reduce its immunogenicity, without changing its binding properties^14–18^ (see Fig. 1a).

Similarity between antibodies can be measured in different ways. The most common ones are via sequence identity or structural similarity^19,20^. With a protein’s function being preserved in the structure, structural similarity is often superior for finding proteins with analogous functions, such as antibodies binding the same epitope^18,21^. However, with orders of magnitude more sequence data available than structural data, a sequence identity search enables the exploration of a much larger space. Available sequence data is also more diverse, as next generation sequencing of B-cell receptors (BCR) is routinely being applied to study adaptive immunity, generating sequences from a range of species^22–24^ and from individuals with differing disease states^25,26^. Furthermore, continuous improvements in high-throughput sequencing methods and increased adoption by research labs means that the amount and diversity of sequence data is rapidly increasing^9,27^. A current limitation of high-throughput sequencing is the possible length that can be sequenced. Most studies therefore only sequence the genomic DNA or RNA of the VH and VL domains instead of the full antibody sequence^9,27,28^. However, as most of the variability and the binding site is located in these regions, this data is still extremely useful for immune repertoire mining.

Whilst freely available, searching this immune repertoire data for similar antibody VH and VL protein sequences, still requires extensive post-processing of each source, such as translating the nucleotide sequences to protein sequences. A database providing a single entry to already processed antibody data to search against is therefore advantageous. One such effort is the Observed Antibody Space (OAS)^29,30^ database, which collates, cleans, annotates, and translates data from publicly available BCR sequencing studies and as of January 2023 contains sequences of the V domain for $\sim$2.4 billion unpaired heavy and light antibody chains. These sequences are derived mostly from humans and mice, but also include sequences from rabbits, rats, rhesus’, camels and humanized mice. While the size of OAS is promising from a scientific perspective, its scale and continuous growth, visualized in Fig. 1b, make mining it effectively a challenge. Though sequence identity calculations are simple, without software specially optimised for the task, the computational cost of exhaustively searching OAS or any other large antibody sequence databases is becoming prohibitive. There is therefore a need for specialized tools to search this space now and in the future.

There exist many tools for searching large datasets of protein sequences for similar sequences, for example BLASTp^31^, CD-HIT-2D^32^, and newer methods such as MMseqs2^33^. However, these tools are all designed around searching a diverse set of proteins and not specifically antibody sequences. BLASTp finds similar sequences by searching for high-scoring 3-mers for a query within a set of target sequences. This scoring is done using a substitution matrix, such as BLOSUM62^34^. For target sequences with exact matched 3-mers, the alignment is then extended in both ends until the score decreases and ranked based on their expect value. BLASTp is much faster than performing a pairwise alignment with the Smith-Waterman algorithm between the query and each target sequence; however, BLASTp does not guarantee optimal alignments^31^. To further increase speed, both CD-HIT-2D and MMseqs2 use fast prefiltering steps to remove target sequences with low identity to the query, thereby reducing the number of pairwise alignments to make, a computational expensive step. CD-HIT-2D prefiltering removes target sequences that have an estimated similarity to the query below a specified threshold. Simplified, the estimation is based on two sequences of certain lengths requiring to share a minimum number of k-mers of different sizes, in order to be above a specific sequence identity. After prefiltering, pairwise alignment is performed on the remaining sequences^35^. Prefiltering with MMSeqs2 is also based on comparing k-mers between the query and target sequence. However, instead of exact matches like CD-HIT-2D, MMseqs2 uses a BLAST-like approach of matching k-mers with a BLOSUM62 score above a certain threshold. For a query and target sequence pair with two k-mer matches found on the same diagonal, an ungapped, and finally a gapped alignment, using Smith-Waterman^36^, is performed^33^.

While prefiltering greatly speeds up sequence search algorithms, it can cause issues when searching a set of closely related sequences, as is the case with antibodies, as the prefiltering step can remove good hits. Further, each tool uses an alignment method designed for general protein sequences, which can result in unreliable antibody alignments, especially in the highly variable CDRs. Within the immunoinformatics field, this alignment problem is often overcome by using antibody specific numbering schemes, like the ImMunoGeneTics (IMGT) scheme^4,37^. Another issue with non-antibody specific tools, is the lack of flexibility in their searches. These tools can only readily be used for searching against the whole antibody chain of target sequences and not for finding similar sequences based on specific subregions. Searching for identical regions at specific antibody positions, especially the CDRs, is often used when looking for similar binders^17^. With the majority of the residues involved in binding being located in the CDRs, the sequence identity over these regions is often more relevant than that of the whole antibody. For some applications, the exact set of residues involved in the paratope may be known. In these cases, searching based on the sequence identity of the paratope may be even more informative (see Fig. 1c). An antibody specific tool that utilizes antibody numbering schemes for better searches, without prefiltering for an exhaustive search, and with the ability to search user-defined continuous or non-continuous regions, such as the paratope, would improve our ability to make best use of the antibody sequence data available.

Recent efforts to create antibody specific searching tools include iReceptor^38^, AbDiver^39^ and CompAIRR^40^. iReceptor, only allows for a V-, D-, or J-gene search or an exact CDR3 match search. AbDiver uses an antibody numbering scheme to align sequences and allows for both CDR3 and whole V domain searches. AbDiver restricts CDR3 searches against CDR3s with a specified V gene and species of origin, and whole V domain searches against sequences with same length CDR1 and 2 and ±1 length CDR3. These restrictions narrow and greatly speed up the search but can occasionally lead to it finding no matches. Further, both iReceptor and AbDiver are not open-source and are only freely available to use via their website, so can only be used against their own databases. While CompAIRR is designed for finding the overlap of CDR3’s across different antibody repertoires, it can also only be used to search for either exact or similar CDR3’s. However, like iRecepter and AbDiver, the restriction of the search limits its use cases, for example none of the tools can search for exact or similar CDR1 or 2, or combinations of CDRs. There therefore exists the need for an open-source antibody specific tool not limited by either being low-throughput, non-exhaustive, or only searching against entire V domain sequences.

Here, we introduce Known Antibody Search (KA-Search), a tool that allows for rapid amino acid sequence identity search across the VH and VL domains of billions of unpaired antibody chains, across either the whole domain, the CDRs, or a user defined antibody region. We demonstrate KA-Search can be used to find the most similar sequences from the $\sim$2 billion heavy chain sequences in the OAS database within 30 min using 5 CPUs. We also show how KA-Search can be used for immune repertoire mining to obtain new insights about an antibody of interest. KA-Search is freely available at https://github.com/oxpig/kasearch.

## Results

Immune repertoire mining to find similar antibodies with shared properties is becoming increasingly computational expensive because of the increase in available antibody sequences. This is illustrated in Fig. 1b, which shows how publicly available sequences in OAS have increased by 1.8 billion in less than four years. Below we describe KA-Search, a freely available tool to search immune repertoires that is optimised to handle the vast amount of available data.

### Computational speed of KA-Search

**Figure 2:**
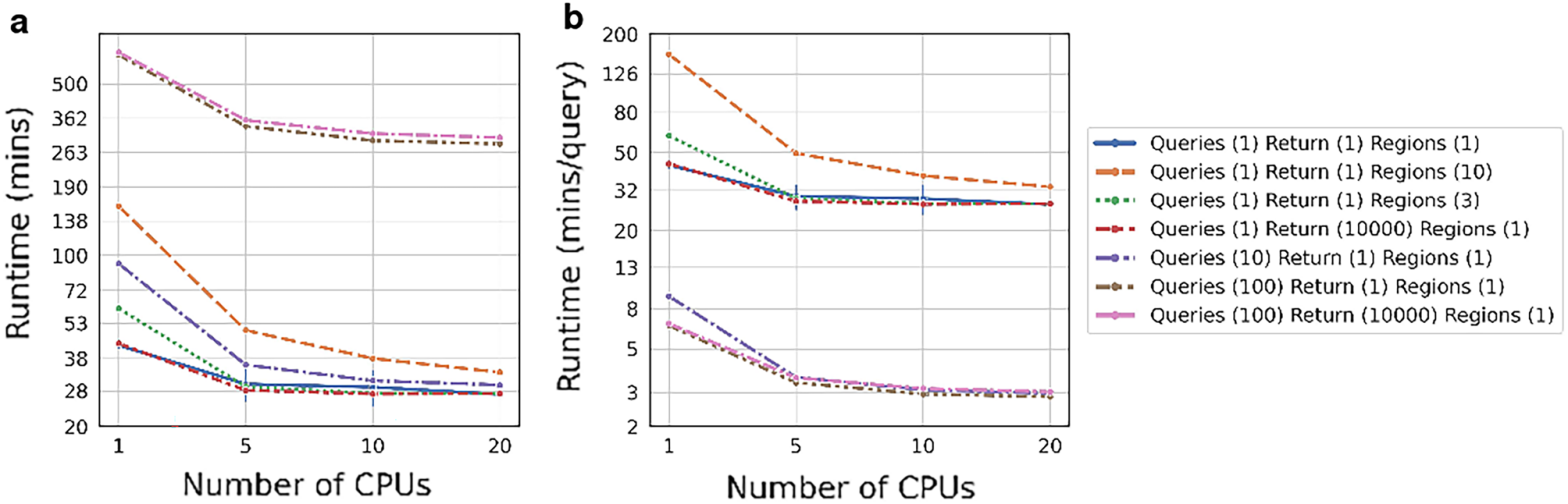
Runtime comparison of KA-Search with different numbers of queries, returned sequences and number of regions. Each search was done against the 2070 million heavy chains in the Observed Antibody Space database. (**a**) The runtime is minimally impacted by the number of closest matches returned, and increases when searching over multiple regions simultaneously or with multiple queries. (**b**) Searching with multiple queries greatly reduces the runtime per query, up until 10 queries per search. It is therefore optimal to search with many queries simultaneously.

KA-Search’s exact speed is dependent on the hardware used, the number of queries, number of output sequences desired and number of regions searched over. Figure 2 shows a comparison between different KA-Search runs with different numbers of CPUs, when searching against the 2070 million heavy chains in OAS-aligned. The number of closest matches returned has a minimal impact on speed, with returning the best or 10,000 best matches taking approximately the same time. Searching over multiple regions simultaneously slows the search but is faster than doing them individually. When using a single CPU one region takes 43.01min ± 9s, three 60.85 min ± 13 s, and ten 158.45 min ± 2s. The time required per query is reduced when searching with multiple queries at a time, as searching with a single query takes $\sim$43 minutes while searching with 100 queries takes $\sim$6.6 min per query. KA-Search is limited by loading data into memory when searching with few queries. The optimal use of KA-Search is therefore to search with many queries and multiple regions simultaneously using multiple CPUs.

### Comparison with other common sequence identity search tools

**Figure 3:**
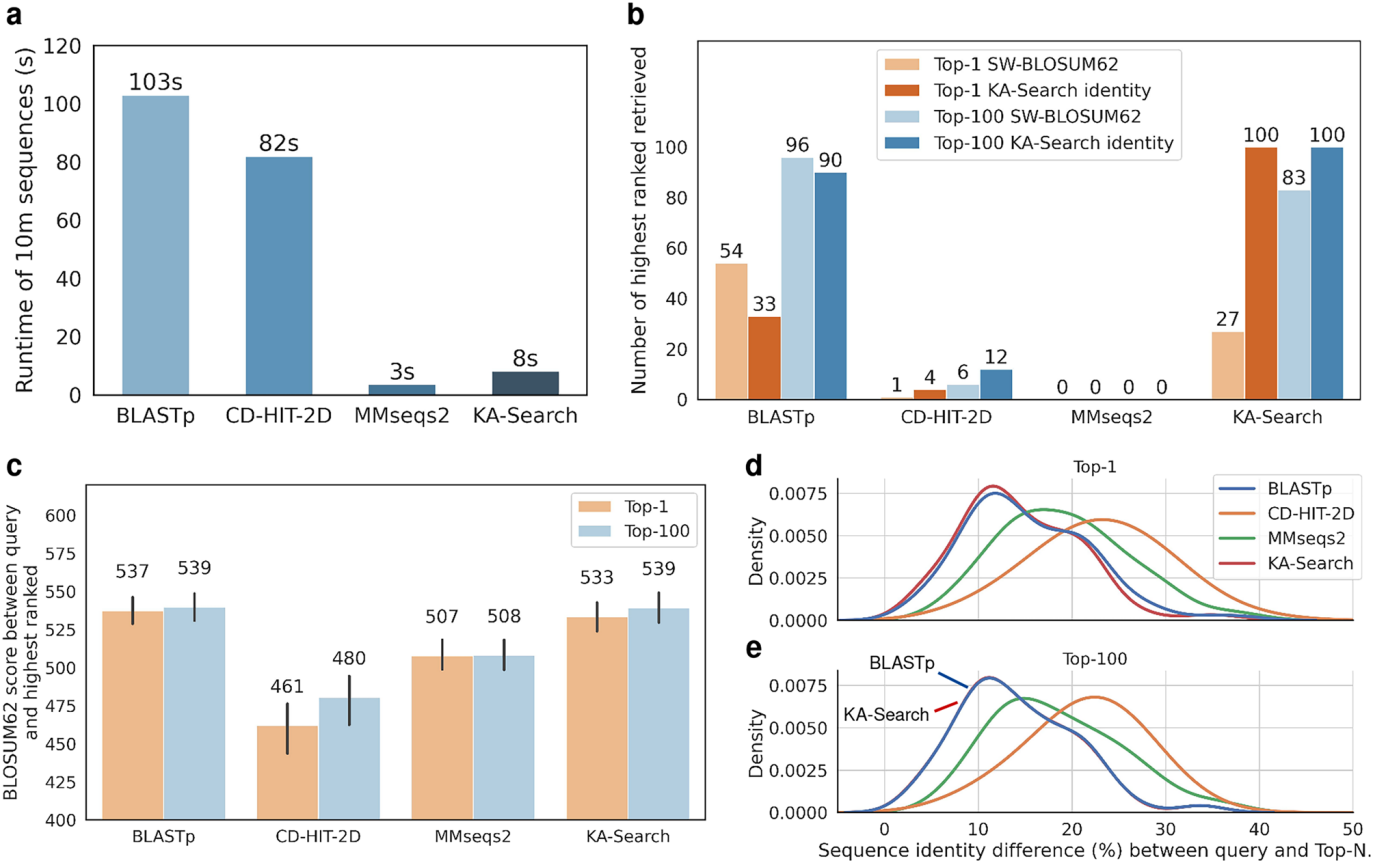
Speed and sensitivity comparison between KA-Search and the commonly used protein search tools BLASTp (version 2.13.0)^31^, CD-Hit-2d (version 4.8.1)^32^ and MMseqs2 (version 13.45111)^33^. The default settings were used for each tool, and each search was done against the whole variable domain. BLOSUM62 scores were calculated by aligning with the Smith-Waterman algorithm using BLOSUM62^34^ as the substitution matrix and gap costs of 11 for existence and 1 for extension. (**a**) Runtime was calculated as the average time it took to search 10 million sequences (OAS-test) with a single query on a single CPU. Sensitivity was compared by the tools ability to find the exact closest match and highest similarity match. (**b**) Sensitivity was calculated by how often each tool returned the closest or the closest within the top-100 match for 100 heavy variable domains (test queries) against the same 10 million sequences. The closest match was defined using two different identity metrics, the highest BLOSUM62 score and KA-Search identity. (**c**) Highest similarity, was first compared with the average BLOSUM62 score between the test queries and target sequences from the top-1 and the closest within the top-100 returned sequences from each search tool. Highest similarity was then compared using the density of sequence identity difference between the test queries and, (**d**) the top-1 returned sequences and, (**e**) the closest within the top-100 returned sequences from each search tool. For top-100, the density of BLASTp and KA-Search are nearly perfectly overlapping. Sequence identity was calculated as the percentage of exact matches after alignment with the Smith-Waterman algorithm using BLOSUM62 as the substitution matrix and gap costs of 11 for existence and 1 for extension.

To compare KA-Search with current freely available and downloadable protein sequence search tools, we selected the amino acid sequence of the VH domain for 100 non-redundant heavy chains of therapeutics, and searched for the most similar sequence within OAS-test, a set of 10 million VH antibody sequences (see methods), using BLASTp^31^, CD-HIT-2D^32^, MMseqs2^33^ and KA-Search. For each tool, the mean and standard deviation of their speed was calculated based on seven runs (see Fig. 3a). KA-Search takes 8.3 s ± 22.7 ms, which is far faster than BLASTp and CD-HIT-2D, 103 s ± 75.9 ms and 82 s ± 55 ms seconds respectively, but slower than MMseqs2 at 3.57 s ± 78.8ms.

In terms of sensitivity, we examined the ability of these sequence search methods to identify the most similar antibody sequence in OAS-test as defined by either the Smith-Waterman aligned BLOSUM62 score used by BLASTp, or the KA-Search identity (see Fig. 3b). Tools that use prefiltering struggle to find the exact closest match. CD-HIT-2D’s highest ranked sequences matched the target sequence with the best BLOSUM62 score and KA-Search identity for only one and four out of the 100 sequences, respectively, and MMseqs2’s highest ranked sequences matched none of the closest target sequences for either metric. When looking for the closest match within the top-100 highest ranked sequences, CD-HIT-2D found the closest match based on the BLOSUM62 score and KA-Search identity for 6 and 12 sequences, respectively, while MMseqs2 found none. BLASTp and KA-Search find the closest match as highest ranked based on the BLOSUM62 score for 54 and 27 sequences, respectively, and for 33 and 100 sequences based on the KA-Search identity. Within the top-100 highest ranked sequences, BLASTp and KA-Search find the closest match for 96 and 83 sequences, respectively, and 90 and 100 sequences based on the KA-Search identity. The full sequence of the test queries and their respective top-1 sequences for each method can be found as Supplementary Data 1 online.

We further compared the average BLOSUM62 score between the query and highest ranked (see Fig. 3c). For BLASTp, CD-HIT-2D, MMseqs2 and KA-Search the score was for the highest ranked on average 537, 461, 507 and 533, respectively, and for best within top-100, on average 539, 480, 508 and 539. Furthermore, we compared the sensitivity of each method by calculating how similar the returned sequences are to the query. This comparison is shown in a density plot of the difference in sequence identity, based on exact matches, between the query and highest ranked sequences (see Fig. 3d,e). For BLASTp, CD-HIT-2D, MMseqs2 and KA-Search this difference was on average 15.26%, 23.04%, 20.29% and 14.43% identity, respectively. The difference between the query and the best within the top-100 highest ranked sequences were 14.12%, 21.41%, 19.52% and 14.06%, respectively.

While CD-HIT-2D is better than MMseqs2 at finding the closest match, MMseqs2 returns on average better matches. The highest ranked from BLASTp and KA-Search are slightly biased towards their used metric; however, the closest match within the top-100 from both methods are very similar. Unlike all the other methods KA-Search is exhaustive, so it finds the exact closest match every time using the KA-Search identity.

### Immune repertoire mining with the COVOX-253 antibody

**Figure 4:**
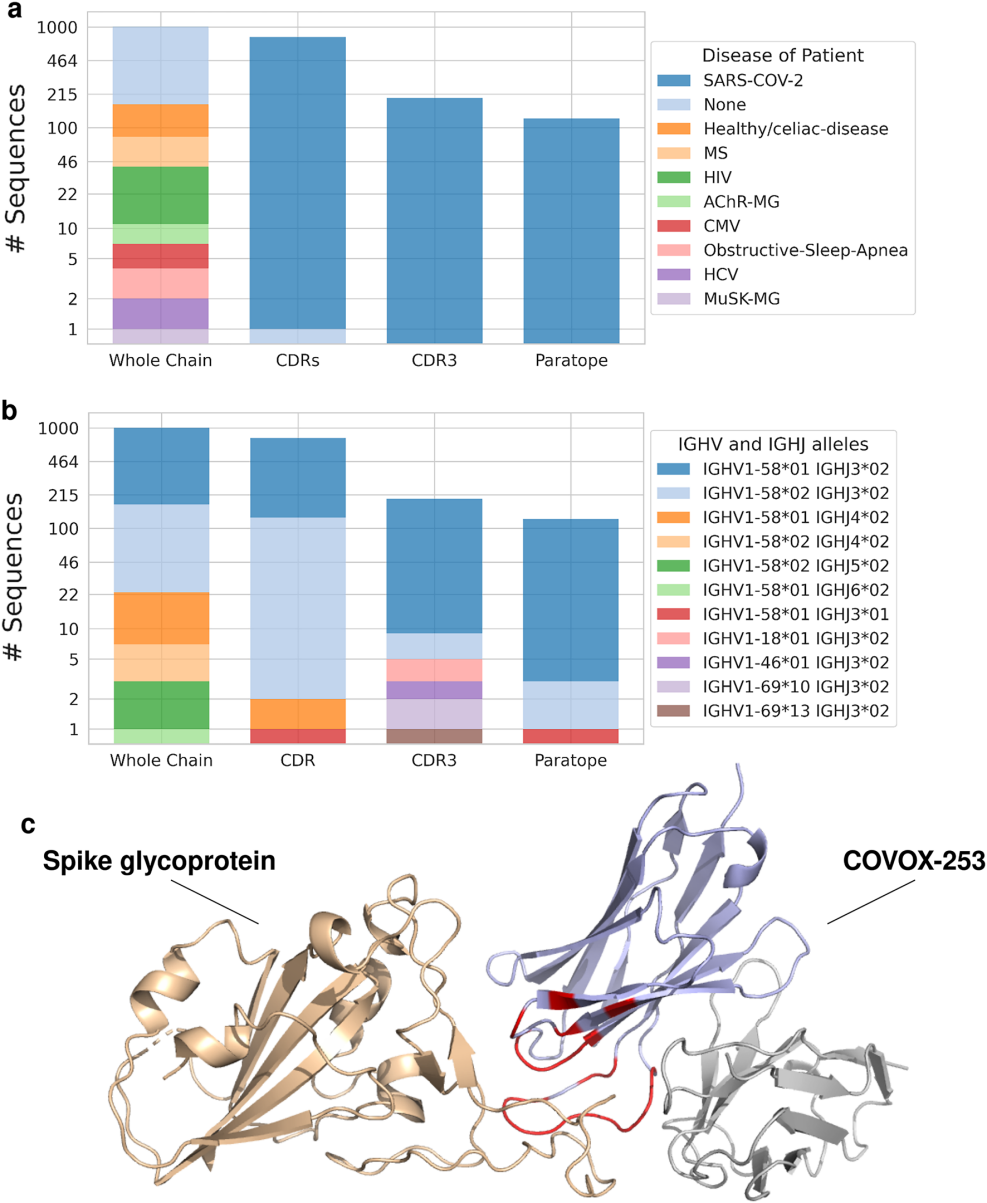
A KA-Search for sequence matches to the heavy variable domain of the SARS-CoV-2 RBD binding antibody COVOX-253. Returned antibodies with over 90% identity across four different regions were visualised based on, (**a**) the disease state of the patient and, (**b**) which V- and J-genes the antibody sequence is derived from. The y-axis is scaled logarithmically to better visualise the data. **c**, The variable domain of COVOX-253’s heavy (purple) and light (grey) chain with the bound spike glycoprotein (beige), derived from PDB 7BEN. The paratope of the heavy chain, which was used to search with KA-Search, is shown in red.

COVOX-253 is an antibody which binds to the neck of SARS-CoV-2’s Receptor-Binding Domain (RBD)^41^. Using KA-Search, up to the 1000 closest sequences to the heavy chain of COVOX-253, with over 90% identity, were extracted for four different regions: the whole V domain, the three CDRs, the CDR3 and the paratope. The paratope was derived from the PDB structure 7BEN and defined as any residue in the antibody which was within 4.5Å of the RBD^42^. Figure 4a shows the disease of the patient the antibody sequence found in OAS comes from and in Fig. 4b each antibody’s combination of V and J genes. Most matched sequences are derived from the gene alleles IGHV1-58*01 and IGHJ3*02; however, COVOX-253’s CDR3 is seen with six different V gene alleles, IGHV1-58*01, IGHV1-58*02, IGHV1-18*01, IGHV1-46*01, IGHV1-69*10 and IGHV1-69*13.

Searching for the closest match using the whole V domain returns 822 sequences from healthy individuals and 178 from patients with one of nine different diseases, eight which are SARS-CoV-2. Searching with the CDR positions returns one sequence from a healthy individual and 789 sequences from patients with SARS-CoV-2, while searching with the CDR3 or paratope positions returns 197 and 124 sequences, respectively, all from SARS-CoV-2 infected patients. The fact that OAS-aligned only contains $\sim$84 million heavy chains from patients with SARS-CoV-2, equivalent to $\sim$4% of all heavy chains in OAS-aligned, highlights the importance of being able to search over specific regions.

## Discussion

Immune repertoire mining is a powerful method for identifying antibodies in nature which are similar to an antibody of interest and can help indicate likely specificity or immunogenicity. However, the number of available antibody sequences are now in the billions and is continuing to grow. Therefore, repertoire mining for highly similar sequences has become increasingly computationally expensive. Existing approaches are limited by either being low-throughput, inaccessible for large scale searches, non-exhaustive, not antibody specific, or only searching against entire V domain sequences. There is therefore a need for a specialized tool, optimized for a rapid and exhaustive search of any antibody region against all known antibodies, to better utilize the full number of available repertoire sequences.

In this paper, we introduce Known Antibody Search (KA-Search), a platform independent antibody search tool. KA-Search finds antibody sequences with an accuracy comparable to BLASTp, while being over an order of magnitude faster and allowing searches over specific regions. KA-Search exploits antibody numbering, allowing us to pre-align antibody sequences to a fixed-length vector. This circumvents pairwise alignment during search, an otherwise time-consuming step. This was done to keep the alignment short and increase speed. The increased speed allows KA-Search to avoid prefiltering and be exhaustive while still retaining a competitive speed. Avoiding prefiltering is crucial, as current prefiltering techniques greatly reduce sensitivity when searching highly related proteins, such as antibodies, where a single mutation can be of great importance. While pre-aligning the antibody sequences increases search speed, the initial pre-alignment is slow. We therefore provide a pre-aligned dataset of the current OAS, ready to use for searching. This dataset can be extended with future OAS updates or in-house data without the need to re-align the existing sequences. A guideline for preparing custom data for search with KA-Search is available at https://github.com/oxpig/kasearch.

Pre-aligning sequences also opens new use-cases. Instead of only searching against the whole antibody V domain, searches can now be focused on specific positions in the alignment. Searches can be specific for the CDRs or regions specific for individual antibodies, such as the paratope. This flexibility allows for studies which were previously difficult to execute. As an example, previously an extensive study was needed to search OAS across the whole V domain, CDRs and CDR3 for the closest match to a set of 242 therapeutics^19^. The same study can now be done on the 804 therapeutics within Thera-SAbDab^43^ (as of August 2022) with KA-Search in less than two days compute and little configuration (see Supplementary Fig. S1 and Supplementary Data 1). KA-Search can also extract the metadata from OAS for the matched sequences, which can be used to obtain new insights about an antibody of interest. Using KA-Search to find the closest sequences with or above 90% identity across four different regions for the SARS-CoV-2 RBD binding COVOX-253 demonstrates the power of searching across particular regions. The closest matches from searching with the whole V domain comes from healthy patients or patients with a variety of diseases. However, binding region specific searches only return sequences found in SARS-CoV-2 infected patients. These sequences could therefore also have RBD binding properties and are possible candidates for further affinity studies. Investigating the genes of the closest sequences, also potentially indicates which other frameworks a region of interest could exist on. For COVOX-253, the CDR3 is seen in sequences with six different V genes, which each could be possible framework candidates for the CDR3. The ability to return high numbers of close matches without decreasing speed, also opens up KA-Search as a means for creating multiple sequence alignments of similar antibody sequences.

A limitation of KA-Search, is that the current version only searches across the V domain, disregarding the constant domain. This is driven by the current very limited number of available sequences of the constant domain. KA-Search can also only search with and against sequences which can be numbered and aligned with the 200 unique positions in the canonical alignment described in the methods. The 0.2% of sequences with rare insertions cannot be searched with KA-Search and are excluded from OAS-aligned. The rare insertions are seen across the whole V domain and while most are likely derived from sequencing or ANARCI numbering errors, e.g. the highly unlikely eight residue insertion giving position 81I, some rare insertions can be contributed to limited data of certain species within OAS, e.g. position 112M seen in camel sequences with a CDR3 longer than 37 residues. As currently OAS mainly contains human and mouse sequences, the 200 positions cover those species well but may as described cover less of the sequences derived from other species. With OAS growing, the unique positions can be updated in the future to better handle other species. Sequences that failed to be aligned are also provided in the OAS-aligned download and can be searched using other methods if desired, for example if the sequence of interest contains rare insertions. Further, KA-Search currently only finds similar sequences using sequence identity. While this is sufficient for finding sequences with few mutations, calculating the sequence similarities using a substitution matrix would allow the exploration of more distant matches.

KA-Search can be run on any system with Python, requiring only 6GB RAM for searching OAS-aligned and 2GB RAM for OAS-aligned-tiny. This enables any researcher to readily search for similar sequences. Currently, when searching with few queries, the primary bottleneck related to the speed is the loading of data into memory to search against. For optimal use or large scale studies, KA-Search therefore benefits considerably from searching with many queries simultaneously on a high-performance computer using multiple CPUs.

KA-Search’s speed, exhaustiveness and flexibility allows it to search the vast numbers of antibody sequences now available seamlessly, find viable mutations and gain new insight into antibodies of interest. We therefore believe KA-Search is a useful tool that will allow the antibody community to explore antibodies in new ways. To maximize KA-Search’s possible contribution to the community, KA-Search is open source and freely available at https://github.com/oxpig/kasearch.

## Method

### Data preprocessing

KA-Search pre-aligns the V domain of antibody sequences to a canonical alignment capable of accommodating the most common numbering positions. To do this, every sequence is first numbered with ANARCI^44^ using the IMGT numbering scheme^4,37^ and then converted to a vector of the same length. As our canonical alignment, we use all of the 196 unique positions seen in at least 40,000 different sequences in OAS, as of May 2022, and four additional unique positions seen in therapeutics from Thera-SAbDab^43^. The exact unique positions are given in Supplementary Table S1 and cover around $\sim$99.8% of sequences in OAS. The 0.2% of antibody sequences that contain a rare insertion in their V domain cannot be searched using KA-Search. This set of unusual sequences is provided together with the aligned sequences and can be searched using other methods. Every aligned sequence is accompanied by two index values which can be used to retrieve its metadata.

All amino acid sequences of the antibody VH and VL domains (derived from the sequence_alignment_aa column) in OAS (September 2022) are pre-aligned using this method to generate a dataset ready to be used by KA-Search. This results in over 2,070 million heavy and 355 million light chain sequences. Sequences are split into heavy and light chains, and by species information, e.g. human, mouse, rabbit, rat, rhesus, camel and humanized, allowing for faster specific searches. We call this data set of heavy and light chains OAS-aligned. OAS-aligned also contains sequences that cannot be aligned in files labeled as unusual, for search using other methods. We also built a subset of the heavy chain dataset, OAS-aligned-small, that contains 118 million heavy chain sequences, which was generated by removing sequences containing ambiguous residues or seen less than five times. Further, a smaller subset of 10 million human heavy chain sequences, OAS-aligned-tiny, was built by removing any sequence in OAS-aligned-small not having a residue at position one and removing duplicate sequences. OAS-aligned, OAS-aligned-small and OAS-aligned-tiny, and the code to update the data sets or expand it with an in-house data set is made freely available with KA-Search (https://github.com/oxpig/kasearch).

### Identity calculation

The identity between a region in the query and target sequence is computed as the percentage of identical residues across a specific region, including indels present in only one of the sequences. A region can be either the whole V domain or a set of antibody numbering positions, such as the CDR3. Length matched sequence identity is only calculated if the compared region has the same length in both the query and target sequence. The identity can also be calculated excluding missing residues at the ends of the sequences; however, the default is to include them. By converting the query and target sequences into fixed length vectors, their sequence identity can be calculated using matrix operations. For KA-Search, this is implemented using the heavily optimised library JAX^45^.

To search for the identity of a specific user-defined region, i.e. the CDRs, a list with the desired positions can be specified (see Fig. 1d). These positions need to be one of the 200 unique positions in the canonical alignment. In default mode, KA-Search will search for similar whole V domains of variable length, and the three CDRs and CDR3 regions with exact length match. KA-Search returns for each target sequence, the sequence identity of the defined region and the target sequence’s metadata, sorted by sequence identity. For the OAS derived data, the metadata includes each column from AIRR’s rearrangement schema^46^ and additional columns derived when preparing OAS^30^ with the last column being the sequence identity.

### Sensitivity and speed comparison

KA-Search was compared to BLASTp (version 2.13.0), CD-Hit-2d (version 4.8.1) and MMseqs2 (version 13.45111), for sensitivity and speed at searching for the closest whole antibody chain match. A set of 100 randomly selected non-redundant heavy chains of therapeutics were used to search for the closest sequence within OAS-test, a set of 10 million heavy chain antibody sequences that could be aligned with the KA-Search canonical alignment. The sequences in OAS-test were randomly extracted from the full OAS database, cleaned and reduced as done in^47^. The 100 therapeutic heavy chains and OAS-test are available for download, see Data Availability.

For BLASTp, we first pre-built a BLAST database of OAS-test using makeblastdb. BLASTp was then run using an expect value threshold of 10, word size of 3, BLOSUM62 as the substitution matrix, gap costs of 11 for existence and 1 for extension, with conditional compositional score matrix adjustment and no other filters or masks. For sensitivity comparisons we sorted hits by the expect value. With CD-Hit we searched using a sequence identity threshold of 70% for the global sequence identity and a word length of 5. For MMseqs2, we first pre-computed a sequence database of OAS-test with the createdb and createindex modules. The prepared database was then searched using the easy-search workflow with the default arguments; sensitivity of 5.7, BLOSUM62 as the substitution matrix, gap costs of 11 for existence and 1 for extension. For sensitivity comparison, 300 sequences were allowed to pass prefiltering and were thereafter sorted by sequence identity. Lastly, for KA-Search we used a pre-aligned OAS-test aligned as described above. For sensitivity comparisons, we compared over the whole V domain without length matching. For each tool, speed was calculated using the same single CPU to search for one sequence against OAS-test and sensitivity by how well each tool found the closest sequence in OAS-test to each query. The closest sequence was defined by either having the highest KA-Search identity or the highest BLOSUM62 score among the top-100 returned from each method. The BLOSUM62 score was calculated after alignment with the Smith-Waterman algorithm, using BLOSUM62 as the substitution matrix, gap costs of 11 for existence and 1 for extension. All time measurements in this paper were performed using CPUs from an Intel Xeon Gold 6240 Processor.

## Supplementary Information

Supplementary Information 1.

Supplementary Information 2.

## Data Availability

The data sets generated for the sensitivity comparison in this study can be found in the following Zenodo repository https://doi.org/10.5281/zenodo.7561985. Links for the OAS-aligned, OAS-aligned-small and OAS-aligned-tiny datasets can be found on the associated github https://github.com/oxpig/kasearch.
