# Supplementary material for: KA-Search, a method for rapid and exhaustive sequence identity search of known antibodies
Source: Sci Rep. 2023 Jul 18;13:11612. doi: 10.1038/s41598-023-38108-7 (PMC10354155; doi:10.1038/s41598-023-38108-7)
Supplement: Supplementary file 1 — Supplementary Information 1. [file 41598_2023_38108_MOESM1_ESM.pdf]

Supplementary Information

KA-Search, a method for rapid and exhaustive sequence identity search of known antibodies

Tobias H. Olsen [1,†], Brennan Abanades [1,†], Iain H. Moal [2] and Charlotte M. Deane [1,3,*]

[1] Oxford Protein Informatics Group, Department of Statistics, University of Oxford, Oxford, United Kingdom
[2] GSK Medicines Research Centre, GlaxoSmithKline plc, Stevenage, United Kingdom
[3] Exscientia plc, Oxford, United Kingdom
[†] These authors contributed equally to this work and share first authorship

Corresponding: deane@stats.ox.ac.uk

The canonical alignment

	The canonical alignment's unique positions
**FRW1**	1, 2, 3, 3A, 4, 5, 6, 7, 8, 9, 10, 11, 12, 13, 14, 15, 16, 17, 18, 19, 20, 21, 22, 23, 24, 25, 26
**CDR1**	27, 28, 29, 30, 31, 32, 32A, 32B, 33C, 33B, 33A, 33, 34, 35, 36, 37, 38
**FRW2**	39, 40, 40A, 41, 42, 43, 44, 44A, 45, 45A, 46, 46A, 47, 47A, 48, 48A, 48B, 49, 49A, 50, 51, 51A, 52, 53, 54, 55
**CDR2**	56, 57, 58, 59, 60, 60A, 60B, 60C, 60D, 61E, 61D, 61C, 61B, 61A, 61, 62, 63, 64, 65
**FRW3**	66, 67, 67A, 67B, 68, 68A, 68B, 69, 69A, 69B, 70, 71, 71A, 71B, 72, 73, 73A, 73B, 74, 75, 76, 77, 78, 79, 80, 80A, 81, 81A, 81B, 81C, 82, 82A, 83, 83A, 83B, 84, 85, 85A, 85B, 85C, 85D, 86, 86A, 87, 88, 89, 90, 91, 92, 93, 94, 95, 96, 96A, 97, 98, 99, 100, 101, 102, 103, 104
**CDR3**	105, 106, 107, 108, 109, 110, 111, 111A, 111B, 111C, 111D, 111E, 111F, 111G, 111H, 111I, 111J, 111K, 111L, 112L, 112K, 112J, 112I, 112H, 112G, 112F, 112E, 112D, 112C, 112B, 112A, 112, 113, 114, 115, 116, 117
**FRW4**	118, 119, 119A, 120, 121, 122, 123, 124, 125, 126, 127, 128

Table S1: Overview of the 200 unique positions in our canonical alignment. The positions are based on IMGT numbering of the variable domain [1, 2], however, instead of representing CDR3 gaps with numbers (i.e. 112.1) we use letters (i.e. 112A). We choose all 196 unique positions seen in at least 40.000 different sequences in OAS, as of May 2022, and four additional unique positions seen in therapeutics from Thera-SAbDab [3]. The four additional positions are 3A, 51A, 85C and 85D.

Closest matches to therapeutic antibodies

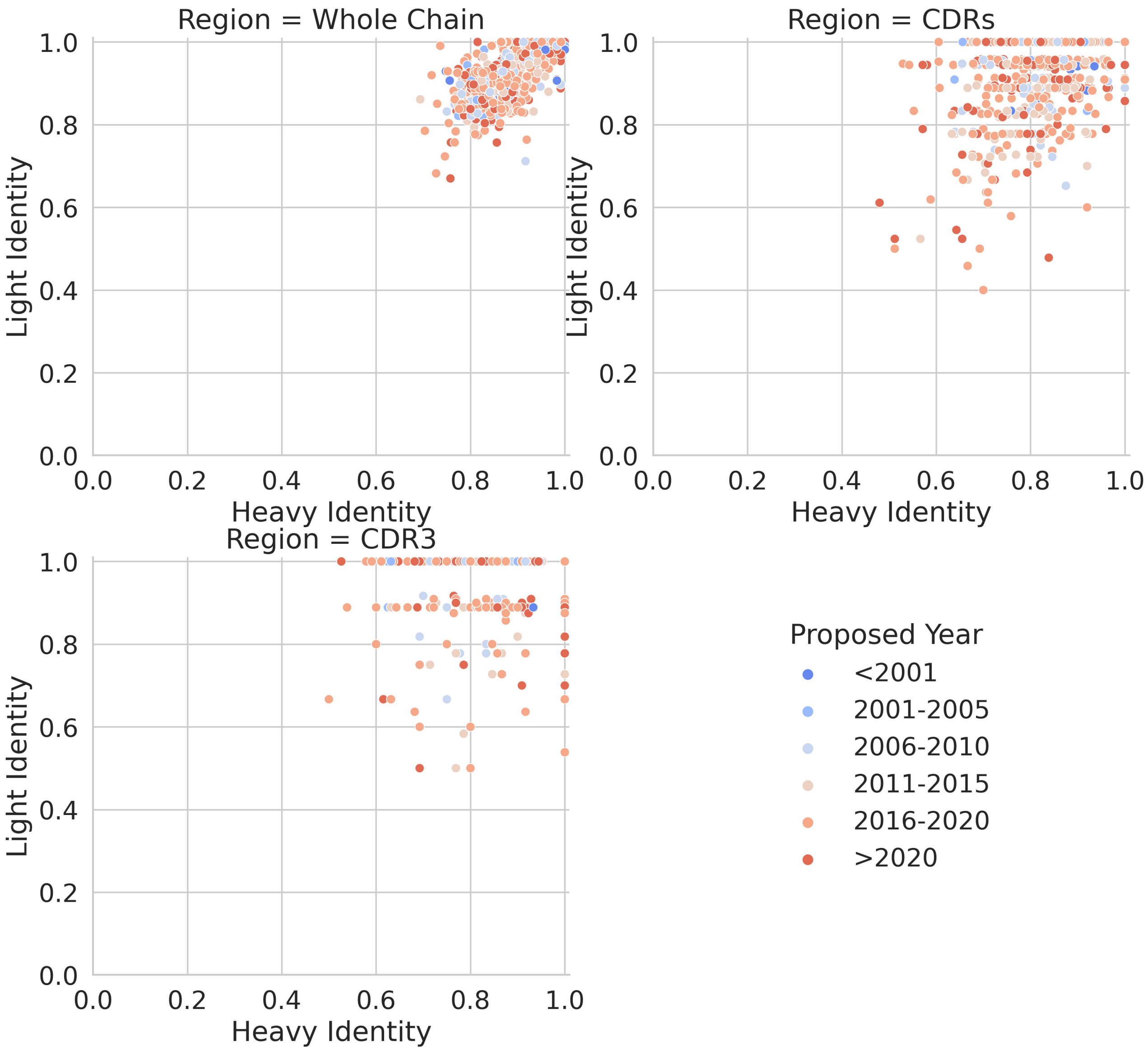

Figure S1: KA-Search was used to find the closest matches in OAS to 804 therapeutics extracted in August 2022 from Thera-SAbDab [3]. Closest matches was found across the whole variable domain, the three CDRs and the CDR3. Each point is colored by the year they were proposed.

References

1. Lefranc, M.-P. Unique database numberings system for immunogenetic analysis. *Immunology Today* **18,** 509 (1997).
2. Lefranc, M.-P., Pommié, C., Ruiz, M., *et al.* IMGT unique numbering for immunoglobulin and T cell receptor variable domains and Ig superfamily V-like domains. *Developmental and comparative immunology* **27,** 55–77 (2003).
3. Raybould, M. I. J., Marks, C., Lewis, A. P., *et al.* Thera-SAbDab: the Therapeutic Structural Antibody Database. *Nucleic Acids Research* **48,** D383–D388 (2020).
